# Comprehensive landscape of the functions and prognostic value of RNA binding proteins in uterine corpus endometrial carcinoma

**DOI:** 10.3389/fmolb.2022.962412

**Published:** 2022-10-03

**Authors:** Yong Yao, Kangping Liu, Yuxuan Wu, Jieyu Zhou, Heyue Jin, Yimin Zhang, Yumin Zhu

**Affiliations:** ^1^ Department of Maternal, Child and Adolescent Health, School of Public Health, Anhui Medical University, Hefei, Anhui, China; ^2^ MOE Key Laboratory of Population Health Across Life Cycle, Hefei, Anhui, China; ^3^ Anhui Provincial Key Laboratory of Population Health and Aristogenics, Anhui Medical University, Hefei, Anhui, China

**Keywords:** uterine corpus endometrial carcinoma, RNA-binding proteins, prognostic model, The Cancer Genome Atlas, survival

## Abstract

**Background:** The dysregulation of RNA binding proteins (RBPs) is involved in tumorigenesis and progression. However, information on the overall function of RNA binding proteins in Uterine Corpus Endometrial Carcinoma (UCEC) remains to be studied. This study aimed to explore Uterine Corpus Endometrial Carcinoma-associated molecular mechanisms and develop an RNA-binding protein-associated prognostic model.

**Methods:** Differently expressed RNA binding proteins were identified between Uterine Corpus Endometrial Carcinoma tumor tissues and normal tissues by R packages (DESeq2, edgeR) from The Cancer Genome Atlas (TCGA) database. Hub RBPs were subsequently identified by univariate and multivariate Cox regression analyses. The cBioPortal platform, R packages (ggplot2), Human Protein Atlas (HPA), and TIMER online database were used to explore the molecular mechanisms of Uterine Corpus Endometrial Carcinoma. Kaplan-Meier (K-M), Area Under Curve (AUC), and the consistency index (c-index) were used to test the performance of our model.

**Results:** We identified 128 differently expressed RNA binding proteins between Uterine Corpus Endometrial Carcinoma tumor tissues and normal tissues. Seven RNA binding proteins genes (*NOP10*, *RBPMS*, *ATXN1*, *SBDS*, *POP5*, *CD3EAP*, *ZC3H12C*) were screened as prognostic hub genes and used to construct a prognostic model. Such a model may be able to predict patient prognosis and acquire the best possible treatment. Further analysis indicated that, based on our model, the patients in the high-risk subgroup had poor overall survival (OS) compared to those in the low-risk subgroup. We also established a nomogram based on seven RNA binding proteins. This nomogram could inform individualized diagnostic and therapeutic strategies for Uterine Corpus Endometrial Carcinoma.

**Conclusion:** Our work focused on systematically analyzing a large cohort of Uterine Corpus Endometrial Carcinoma patients in the The Cancer Genome Atlas database. We subsequently constructed a robust prognostic model based on seven RNA binding proteins that may soon inform individualized diagnosis and treatment.

## Background

Uterine Corpus Endometrial Carcinoma (UCEC) is one of the most prevalent malignant tumors in females, with an estimated incidence of ∼4.4% (Bray et al., 2018). Recently, the incidence of UCEC has gradually increased, which accounts for 20%–30% of all gynecologic malignancies. UCEC is the second most common gynecologic malignancy after cervical cancer (Ascano et al., 2012). Most patients diagnosed with endometrial cancer are postmenopausal, and the median age at diagnosis is 60. However, up to 14% of cases occur in pre-menopausal women, mainly due to an elevated body mass index (BMI) (Garg and Soslow, 2014; Wise et al., 2016).

Five-year survival rates for endometrial cancer vary depending on the stage at diagnosis. Five-year survival is ≥ 95% in patients with tumors confined to the uterus but drops sharply when tumors spread outside the uterus, with survival estimated at 69% in patients with regional metastases and 17% in patients with distant metastases (Creasman et al., 2006; Zanders et al., 2013).

Approximately 90% of patients with UCEC present with early clinical symptoms, such as vaginal bleeding, and about 75% of patients can be diagnosed and treated early. Unfortunately, some patients with early-stage UCEC are at high risk of recurrence, with approximately 18% dying from the recurrent disease (Amant et al., 2005). Therefore, prognostic indicators and models may inform individualized diagnosis and treatment.

RNA binding proteins (RBPs) bind to RNA and regulate RNA function (Zhu et al., 2019). RBPs recruit various factors and enzymes and form different compounds in different combinations to regulate the fate and/or function of the target RNAs (Lukong et al., 2008; Hong, 2017). Dysfunctional RBPs are associated with various human diseases. Central nervous system RNA-binding protein mutations can lead to the aggregation of abnormal proteins that contribute to the progression of various neurodegenerative diseases (Johnson et al., 2018; Duan et al., 2019). Abnormalities in the cardiovascular system RNA-binding proteins may contribute to cardiovascular disease by their effects on a wide range of post-transcriptional events (de Bruin et al., 2017). RBPs are abnormally expressed in tumors, affect the translation of mRNA into protein, and contribute to carcinogenesis (Johnson et al., 2018; Duan et al., 2019). In cancer, abnormal expression of RBPs regulates the expression levels of target RNAs associated with cancer cell proliferation, apoptosis, angiogenesis, senescence, and epithelial-mesenchymal transition (EMT)/invasion/metastasis. RBP-mediated regulation ultimately contributes to cancer development and pathology (Pereira et al., 2017a; Hentze et al., 2018). However, there are relatively few systematic studies of RBP associated with UCEC in gynecological malignancies.

Therefore, we collected UCEC expression profiles and clinical information from the TCGA database to explore the relationships among hub RBPs, clinicopathological characteristics, and prognosis in UCEC patients. We first obtained genes that were differentially expressed between tumor and normal tissues and systematically explored their potential mechanisms. This process produced a seven-RBP model with a high prognostic value. Finally, we developed a clinically applicable nomogram that may be useful to clinicians for clinical diagnosis and prognosis prediction.

## Methods

### Data processing

**FIGURE 1:**
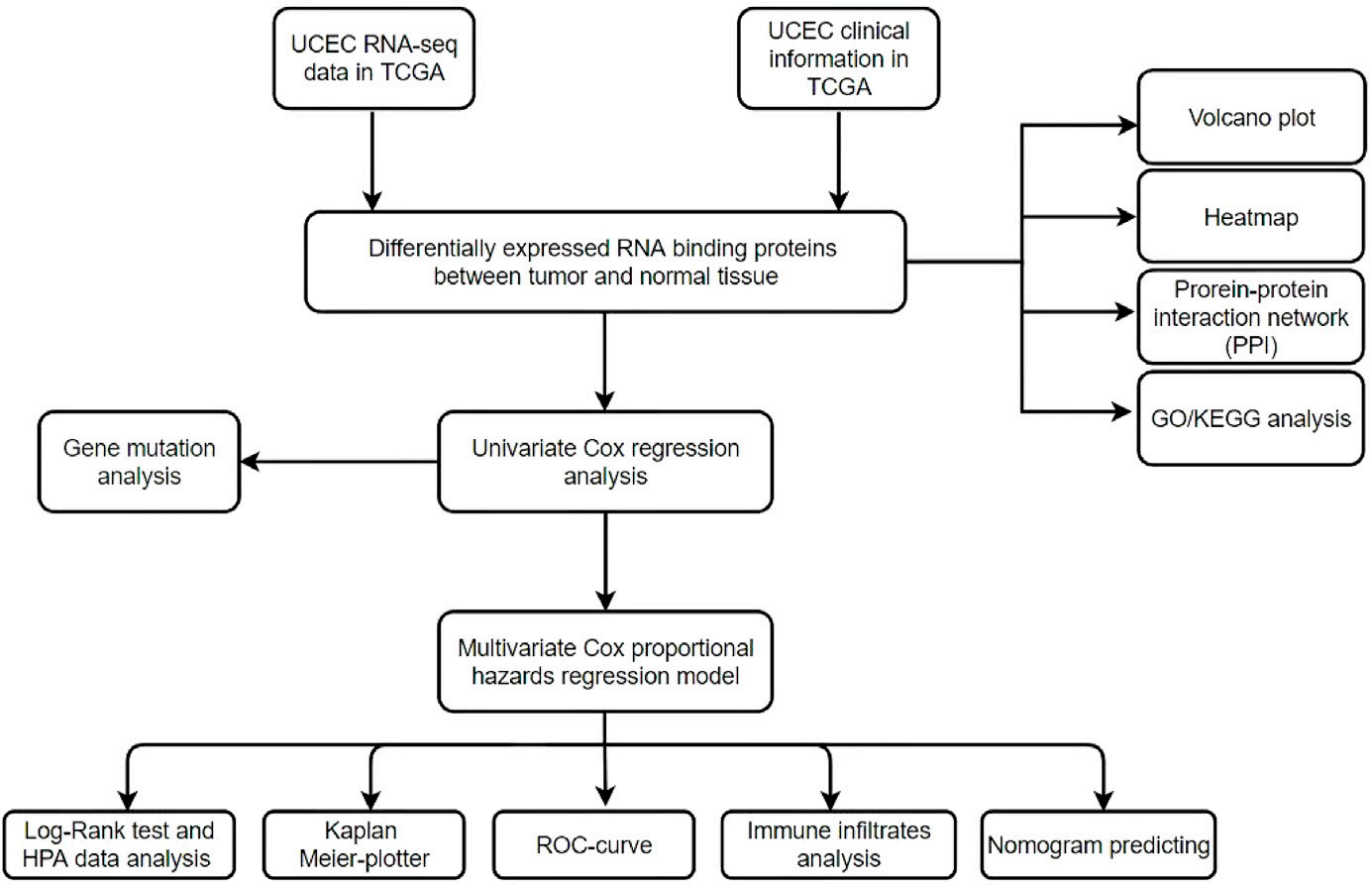
Flowchart for analyzing RBPs in UCEC.

The flowchart of this study is displayed in Figure 1. RNA-seq and clinical information data of UCEC patients were collected from TCGA database with the following steps: 552 UCEC patients with RNA-seq data were included in this study, nine samples with unknown survival time or no survival status were removed. Finally, 543 patients with UCEC and the corresponding clinical information, such as survival information, age, grade and stage were enrolled for further study (Table 1). Then we identified differential expressed genes and got expression matrix from the counts data using R packages (DESeq2 and edgeR). |log2 fold change | > 1 and False discovery rate <0.05 are used as the threshold to screen differently expressed RBPs, these data were used for subsequent analysis.

**TABLE 1:** Demographic characteristics of the study subjects.

Feature	Number
Age(%)	
<50	45(8.3)
≥50	495(91.2)
unknow	3(0.5)
Survival state(%)	
Die	462(85.1)
Alive	81(14.9)
Survival time(%)	
<5 years	435(80.1)
≥5 years	108(19.9)
Tumor grade(%)	
grade1	98(18.1)
grade2	120(22.1)
grade3	314(57.8)
high grade	11(2.0)

Considering the number of normal tissue from TCGA database is less, we then downloaded 142 normal tissue datasets from the GTEx database (https://gtexportal.org/home/) to verify our results.

### GO and KEGG functional enrichment analysis

In order to explore the main biological functions and signaling pathways of differently expressed RBPs, g: GOSt tool at g:Profiler website (https://biit.cs.ut.ee/gprofiler/gost) was used for functional enrichment analysis (Raudvere et al., 2019). The false discovery rate (FDR) < 0.05 was thought to be statistically signifificant. And the results were visualized *via* “GOplot” R package.

### PPI network construction and key module analysis

The differently expressed RBPs were submitted to the STRING database (http://www.string-db.org/) to identify protein-protein interaction. An interaction score of 0.4 (median confidence) was set in the system configuration (Damian et al., 2015; Guerrero, 2020). Analyzing functional interactions between proteins can provide new insights into protein function and help discover functional connections between proteins at the genomic level (Armendáriz-Castillo et al., 2020; López-Cortés et al., 2020). Submit the data to Cytoscape 3.7.1 software for network visualization.

In addition, Cytoscape Plugin Molecular Complex Detection (MCODE) was used to screen out important functional modules of PPI network using score and number of nodes >2 as thresholds.

### Identification and validation of prognostic hub RNA binding proteins

First, univariate Cox analysis was applied to analyze the relationship between the 128 differently expressed genes previously screened and the survival status of the samples in the UCEC cohort in the TCGA database, then 30 RBPs were identified to go for multivariate Cox regression analysis. Ultimately, we identified seven RBPs associated with prognosis. Subsequently, we analyzed the expression of these seven hub RBPs in normal and tumor tissue samples in the UCEC cancer cohort in the TCGA database, and the results showed that the expression of these seven hub RBPs differed significantly, which is also validating that these seven RBPs are critical.

### Calculation of TMB

To calculate the TMB of each UCEC tumor sample and observe the mutations of 30 prognosis-related RBPs in the sample, we selected somatic mutation data from the GDC (https://portal.gdc.cancer.gov/) website and then used the R package “maftools” to realize these 30 prognosis-associated RNA gene mutations in the sample visualization.

### Construction and validation of an RNA binding proteins-Gene prognostic signature

We constructed a risk score signature by using multivariate Cox regression based on the previously obtained RBPs using the survival R package in TCGA. The risk score was calculated by the following formula: Risk score = Expression of gene1 × Coefficient of gene1 + Expression of gene2 × Coefficient of gene2 + … Expression of geneN × Coefficient of geneN (Chen et al., 2007; Wu et al., 2019). To evaluate the performance of the prediction model, we divided all UCEC patients into high-risk and low-risk groups based on the median risk score, and the Kaplan-Meier curve analysis and log-rank test were used to assess the survival difference between two subgroups by “Survival” R package. We also used the “survivalROC” package in R to plot the receiver operating characteristic (ROC) curves and calculate the area under the curve (AUC) values, which was used to evaluate the predictive capacity of this model (Heagerty et al., 2000). We calculated AUC values and its 95% CI at 1-,3-,5-years in the TCGA cohort. To determine the feasibility and reliability of the seven-gene prognostic signature, we validated it by using testing set A (n = 272) and testing set B (n = 271) (Zhou et al., 2020).

### Genetic alteration, and DNA methylation analysis of the seven Prognosis-Related RBP Genes

OncoPrinter was performed to show the frequency of these seven prognosis-related RBPs’ genetic alterations. The gene alteration and DNA methylation data of hub RBP genes in UCEC patients were collected from the cBioPortal platform (http://www.cbioportal.org/). The correlation between copy number variation (CNV), methylation, and mRNA expression is then visualized by using the ggplot2 R package (https://github.com/tidyverse/ggplot2).

### Verification of the expression levels of the hub RBPs

The Human Protein Atlas (HPA) online database (http://www.proteinatlas.org/) was used to investigate the differential expression of the seven hub RBPs at the protein level between tumor and normal tissues.

### Immune infiltration analysis

Tumor infiltrating immune cells can profoundly influence the progress of tumor and the effect of anticancer therapy by promoting tumor and antitumor effects (Balachandran et al., 2015), Therefore, quantification of tumor-infiltrating immune cells is expected to reveal the multifaceted role of the immune system in human cancer, as well as its involvement in tumor escape mechanisms and response to treatment. Tumor Immune Estimation Resource (TIMER) is a comprehensive resource for systematic analysis of various malignant tumors. Based on the TIMER database, we further evaluated the relationship between immune cell types (CD4+T cells, CD8+T cells, B cells, dendritic cells, macrophages, neutrophils and tumor purity) and seven hub RBPs in UCEC.

### Development of a predictive nomogram

We used the independent factors age, tumor grade, tumor stage, tumor infiltration, and risk score identified above as the covariates, along with the “rms” package in R to develop a predictive line graph to predict survival at 1, 3, and 5 years for patients with UCEC. Nomograms are important in the modern medical decision making process because they can help predict the probability of clinical events by integrating different prognostic and deterministic variables to help predict the probability of clinical events (Eberhardt et al., 2015). The discriminative power and predictive accuracy were then assessed using the c-index and calibration curve, metrics for assessing the performance of the nomogram, respectively, in conjunction with decision curve analysis (DCA) to estimate the clinical usefulness and net benefit of the predictive nomogram (Xiang et al., 2020).

### Statistical analysis

Statistical analyses were implemented using R software (version 4.1.3). The calibration curves were used to assess the relationship between the predicted probabilities and actual outcomes, and the calibration was evaluated by bootstrapping 500 times. All p -values of statistical data were based on two-sided statistical tests, and data with *p* < 0.05 was considered to be statistically significant.

## Results

### Exploration of differently expressed RNA binding proteins in uterine corpus endometrial carcinoma

**FIGURE 2:**
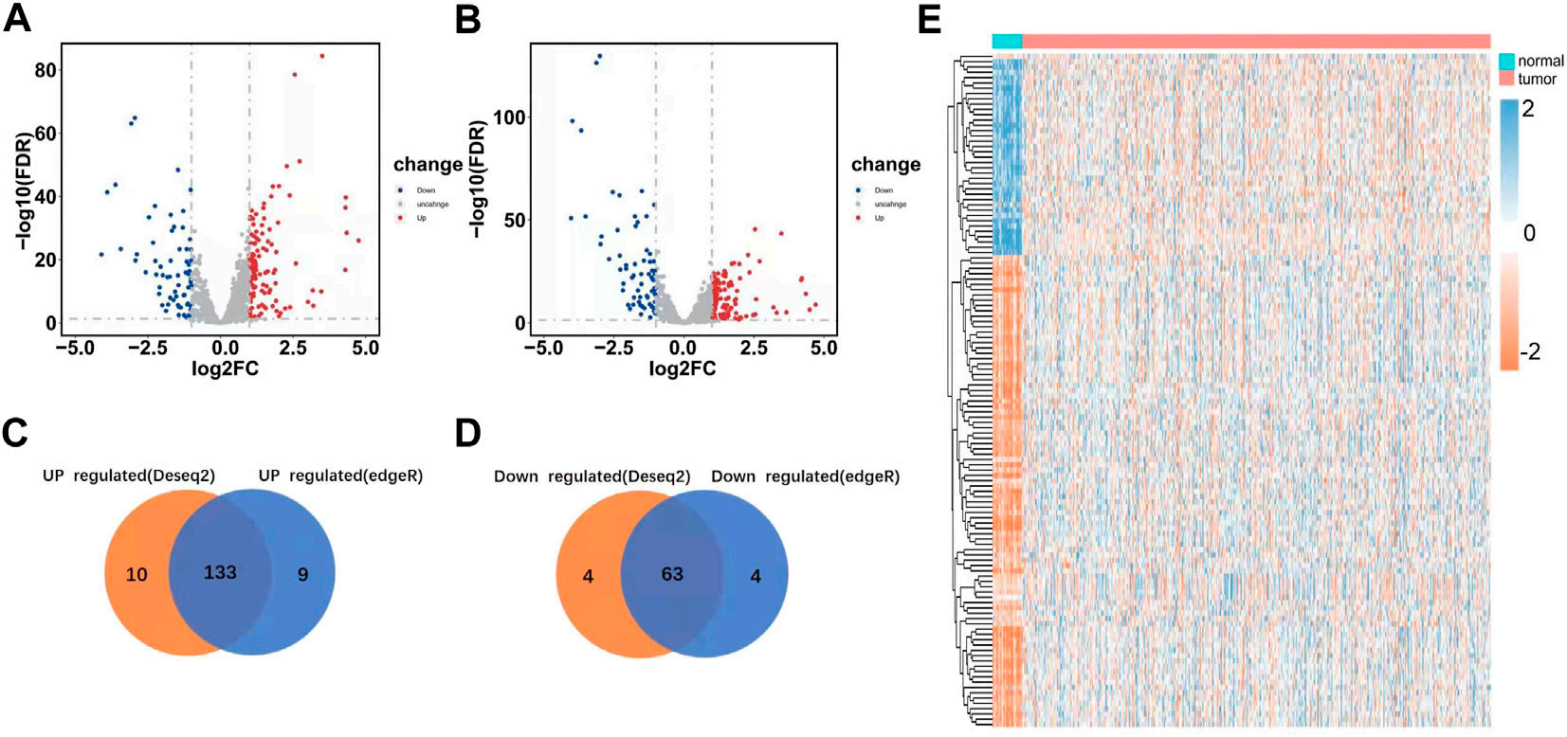
Differently expressed RBPs in UCEC. **(A)** Differently expressed RBPs as screened out by DESeq2 and meet the criteria of |log2FC|> 1, FDR<0.05. **(B)** Differently expressed RBP as screened out by edgeR and meet the criteria of |log2FC|> 1, FDR<0.05. **(C–D)** Venn plots of differently expressed RBP were screened by DESeq2 and edgeR. **(E)** Heatmaps of the differently expressed RBPs.

We systematically analyzed key roles and prognostic values of RBPs in UCEC using several computational methods. UCEC data were downloaded from TCGA and contained 543 tumor and 35 normal tissue samples. The R software packages, which include DESeq2 and edgeR, were applied to handle the data and discover the differently expressed RBPs. A total of 1,542 RBPs were included in the analysis (Gerstberger et al., 2014). Finally, 190 differently expressed RBPs were identified by DESeq2; these contained 123 upregulated RBPs and 67 down-regulated RBPs (Figure 2A). 189 differently expressed RBPs were identified by EdgeR, which contained 122 upregulated RBPs and 67 down-regulated RBPs (Figure 2B). Integrating the results from DESeq2 and edgeR, 176 differently expressed RBP were screened out; 113 were upregulated, and 63 were down-regulated (Figures 2C–E).

### Functional enrichment analysis of differential expression of RNA binding proteins

**FIGURE 3:**
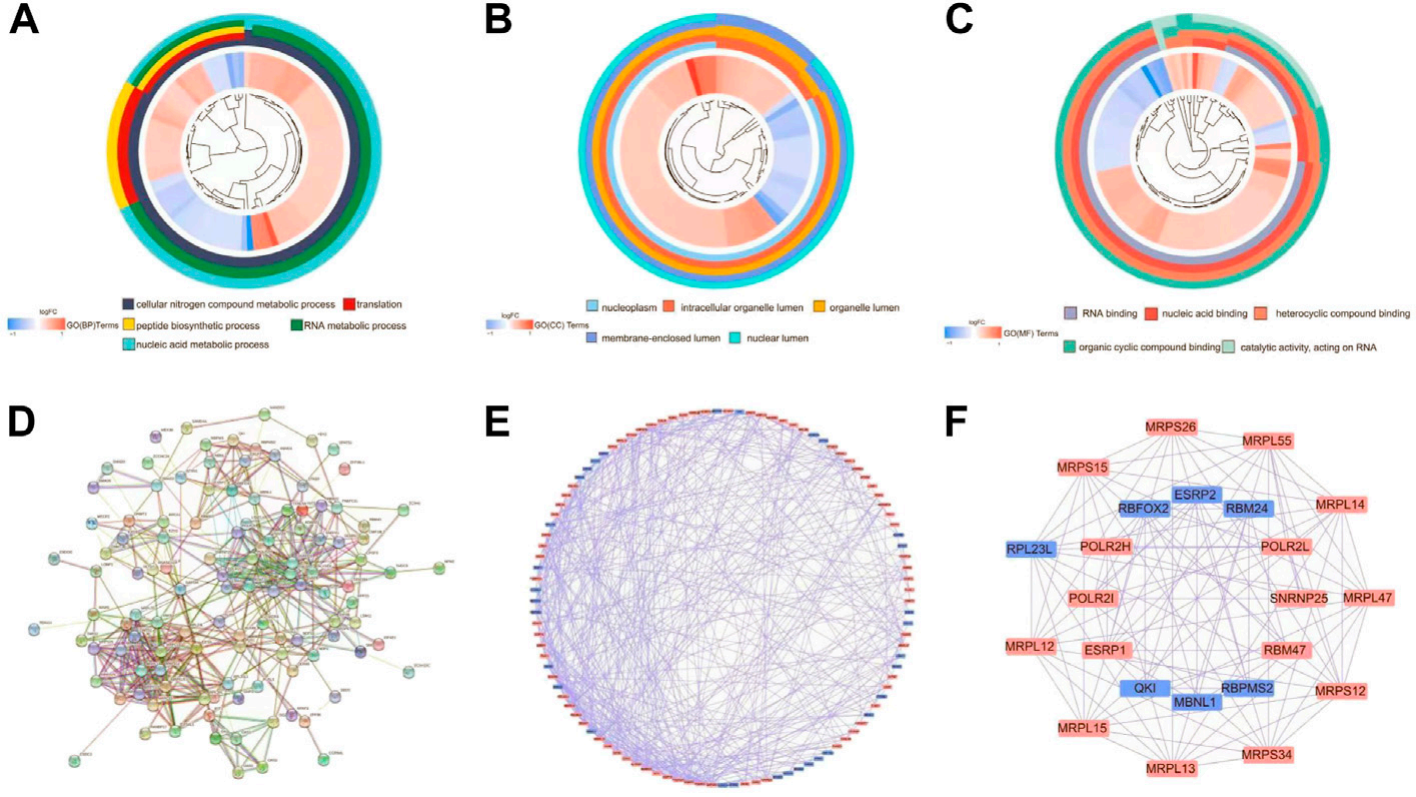
Functional enrichment analyses for differently expressed genes and Protein-protein interaction (PPI) network construction and modules analysis. **(A)** Circos plot showing enriched GO-BP (Gene Ontology-Biological Process) terms of differently expressed RBPs. **(B)** Circos plot showing enriched GO-CC (Gene Ontology-Cellular Component) terms of differently expressed RBPs. **(C)** Circos plot showing enriched GO-MF (Gene Ontology-Molecular Function) terms of differently expressed RBPs. **(D)** PPI interaction network map obtained from STRING website. **(E)** Cytoscape visualizes the genes of the interacting PPI network. Red nodes represent up regulated genes, while blue nodes refer to down regulated genes. **(F)** The most significant MCODE components form the PPI network.

We performed GO and KEGG functional analyses to elucidate the potential biological functions and related mechanisms of differently expressed RBPs. GO analysis consists of a biological process (BP), cellular component (CC), and molecular function (MF). The differently expressed RBPs were mostly enriched in BP: including cellular nitrogen compound metabolic process, RNA metabolic process, and nucleic acid metabolic process (Figure 3A). For CC, DEGs were mostly enriched in the nucleoplasm, intracellular organelle lumen, organelle lumen, membrane-enclosed lumen, and nuclear lumen (Figure 3B). Molecular function analysis showed that DEGs were significantly involved in heterocyclic compound binding, organic cyclic compound binding, nucleic acid binding, and RNA binding (Figure 3C). KEGG pathway enrichment analysis revealed that all differently expressed RBPs were significantly associated with the spliceosome, ribosome, and mRNA surveillance pathways (Supplementary Figure S1).

### PPI network construction and key module analysis

Next, we explored the effects of 128 differently expressed RBPs in UCEC using a PPI network. RBP interaction relationship data were downloaded from the STRING tool and imported into Cytoscape for visualization. The PPI network consists of 105 nodes and 459 edges (Figure 3D). We analyzed the co-representation network using the MCODE plugin to identify key modules, including the two most important modules (Figures 3E,F). GO-BP enrichment analysis showed that RBPs in module one were mainly enriched in the protein metabolic, macromolecule biosynthetic, and cellular protein metabolic processes. RBPs in module two were mainly enriched in RNA splicing, regulation of the mRNA metabolic process, and mRNA processing (Supplementary Figure S2).

### Univariate cox regression to identify prognosis-related RNA binding proteins

**FIGURE 4:**
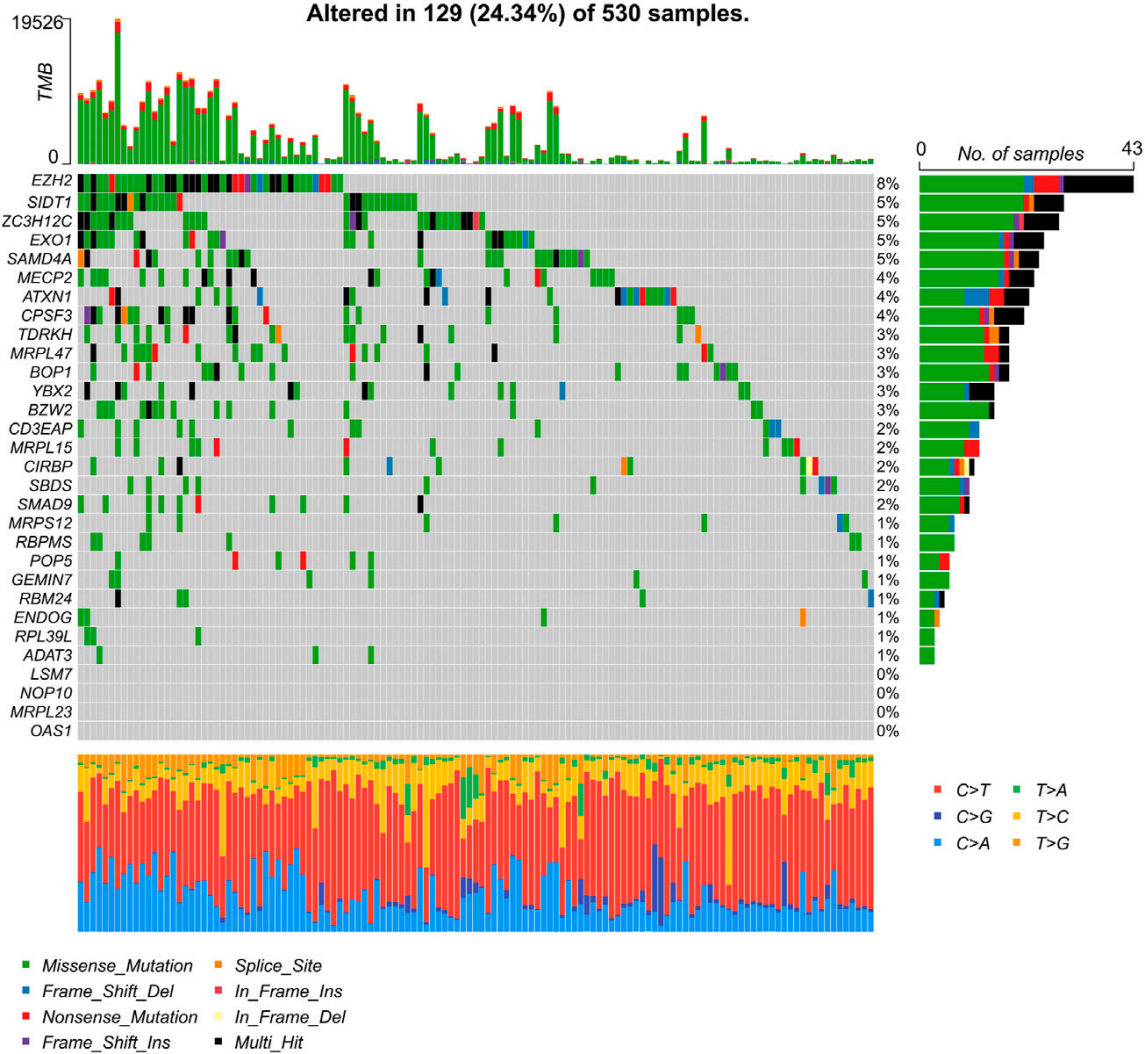
Characterization of 30 prognosis-related RBPs’ tumor mutations in UCEC samples. Mutations of 30 prognosis-related RBPs in UCEC samples, column representative genes.

To establish our novel predictive signature for UCEC patients, we applied univariate cox regression analysis in differently expressed RBPs; RBPs with *p*-values <0.05 in univariate Cox analysis were considered significantly associated with OS, and hazard risk (HR) was calculated to identify risk-increasing (HR > 1) and protective genes (HR < 1). 30 RBPs were defined as prognosis-related RBPs based on these criteria (Table 2). These 30 prognosis-related RBPs were mutated in approximately 24.34% of the total 530 samples, with higher total mutations in *EZH2*, *SIDT1*, and *ZC3H12C* (8%, 5%, and 5%); no mutations were observed in *LSM7*, *NOP10*, *MRPL23*, and *OAS1*. Missense mutations were the most common somatic mutational types (Figure 4).

**TABLE 2:** Unicox results of differential RBPs.

Gene	HR	z	*p*-value
CD3EAP	2.391458923	4.346875367	0.000013809
CIRBP	0.541178543	−3.600328164	0.000317816
LSM7	0.572482401	−3.591732503	0.000328487
YBX2	1.357758771	3.378268126	0.000729439
MRPL15	1.762735328	3.283912261	0.001023768
TDRKH	1.524846953	2.838682482	0.004530021
NOP10	0.596455478	−2.712753596	0.00667267
SAMD4A	1.765905126	2.683683078	0.00728161
SBDS	1.603059021	2.632678292	0.008471455
MRPL23	0.646948615	−2.593175617	0.00950942
MECP2	2.336593091	2.532837714	0.011314332
POP5	0.577808872	−2.487191221	0.012875617
ZC3H12C	1.950542754	2.469430102	0.013532845
MRPS12	1.512001062	2.434556081	0.014910069
ATXN1	0.571110339	−2.427120695	0.015219192
MRPL47	1.435094464	2.384334715	0.017110032
CPSF3	1.812881357	2.377458125	0.017432422
GEMIN7	1.657657963	2.346262128	0.018962766
BOP1	1.442119432	2.303386634	0.021257099
RPL39L	1.238062894	2.298528417	0.021531732
ENDOG	0.725979466	−2.208393206	0.027216875
EZH2	1.433468536	2.206975815	0.027315749
EXO1	1.402591262	2.178166507	0.029393643
RBM24	0.725021991	−2.075614682	0.037929602
SIDT1	0.706953500	−2.064534812	0.038967037
ADAT3	0.710307233	−2.038870497	0.041462954
RBPMS	0.785179077	−2.008192852	0.044622802
OAS1	1.179468782	2.006127294	0.044842668
SMAD9	0.761868713	−2.004805457	0.044983849
BZW2	1.390287208	1.974205930	0.048358332

### Construction and validation of RNA binding proteins prognostic model

**FIGURE 5:**
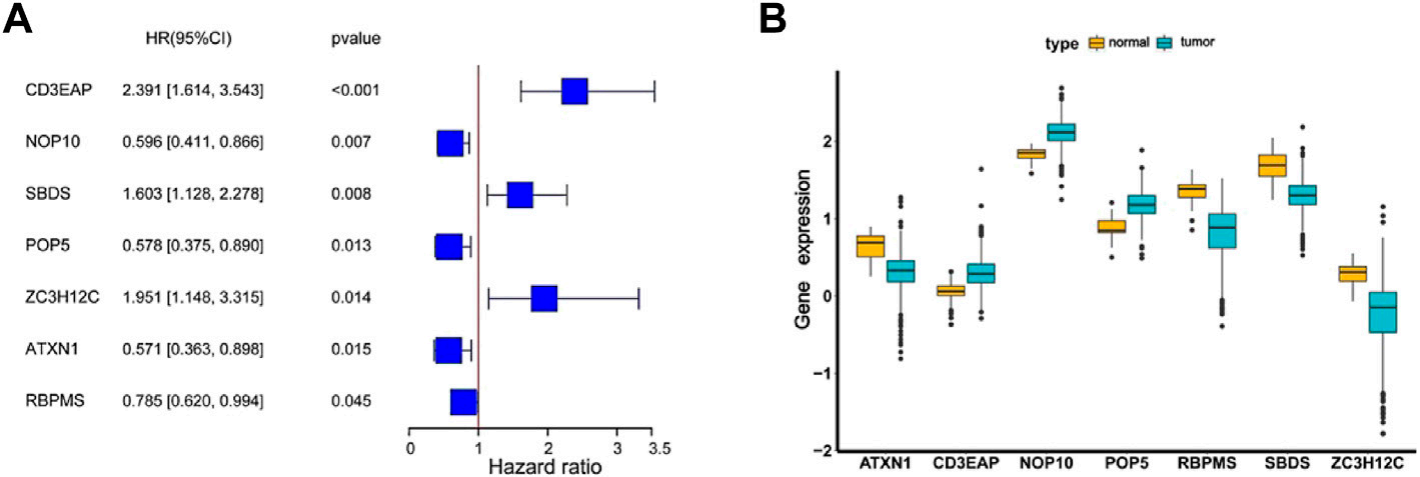
The coefficients and expression profiles of seven hub RBPs. **(A)** The coefficients of seven hub RBPs screened by univariate and multivariate Cox regression analysis. **(B)** The seven differently expressed genes were screened for expression in normal and cancer samples.

The 30 candidate RBPs were then subjected to a multivariate Cox regression analysis. The relationships between all candidate RBPs and survival time were analyzed, and seven hub RBPs associated with prognosis were subjected to further screening. These were *POP5*, *NOP10*, *CD3EAP*, *SBDS*, *ZC3H12C*, *ATXN1*, *RBPMS* (*CD3EAP*, *SBDS* and *ZC3H12C* HR > 1; *NOP10*, *POP5*, *ATXN1* and *RBPMS* HR < 1) respectively (Figure 5A). Compared to normal samples, *ATXN1, RBPMS, SBDS,* and *ZC3H12C* were downregulated, and *CD3EAP, NOP10,* and *POP5* were upregulated in UCEC tumor samples (Figure 5B). Moreover, we collected 142 normal tissue samples from GTEx (Genotype-Tissue Expression) database, then validated the regulation patterns of seven hub RBPs based on 552 tumor tissue samples and 175 normal tissue samples (35 normal tissue samples from TCGA and 142 normal tissue samples from GTEx). *CD3EAP*, *NOP10*, and *POP5* were significantly up-regulated in tumor tissue samples, while *ATXN1*, *RBPMS*, *SBDS*, and *ZC3H12C* were significantly downregulated in tumor tissue samples calculating by DESeq2 and edgeR (Supplementary Figure S3), which were consistent with our previous results (Figure 5B).

The seven hub RBPs chosen from the multiple Cox regression method were used to establish the predictive model. The risk score of each patient was calculated based on the coefficients: h(t) = h0(t)exp (0.7039*CD3EAP-0.3941*NOP10 + 0.3044*SBDS-0.3529*POP5+0.6049*ZC3H12C-0.6804*ATXN1-0.1991*RBPMS).

**FIGURE 6:**
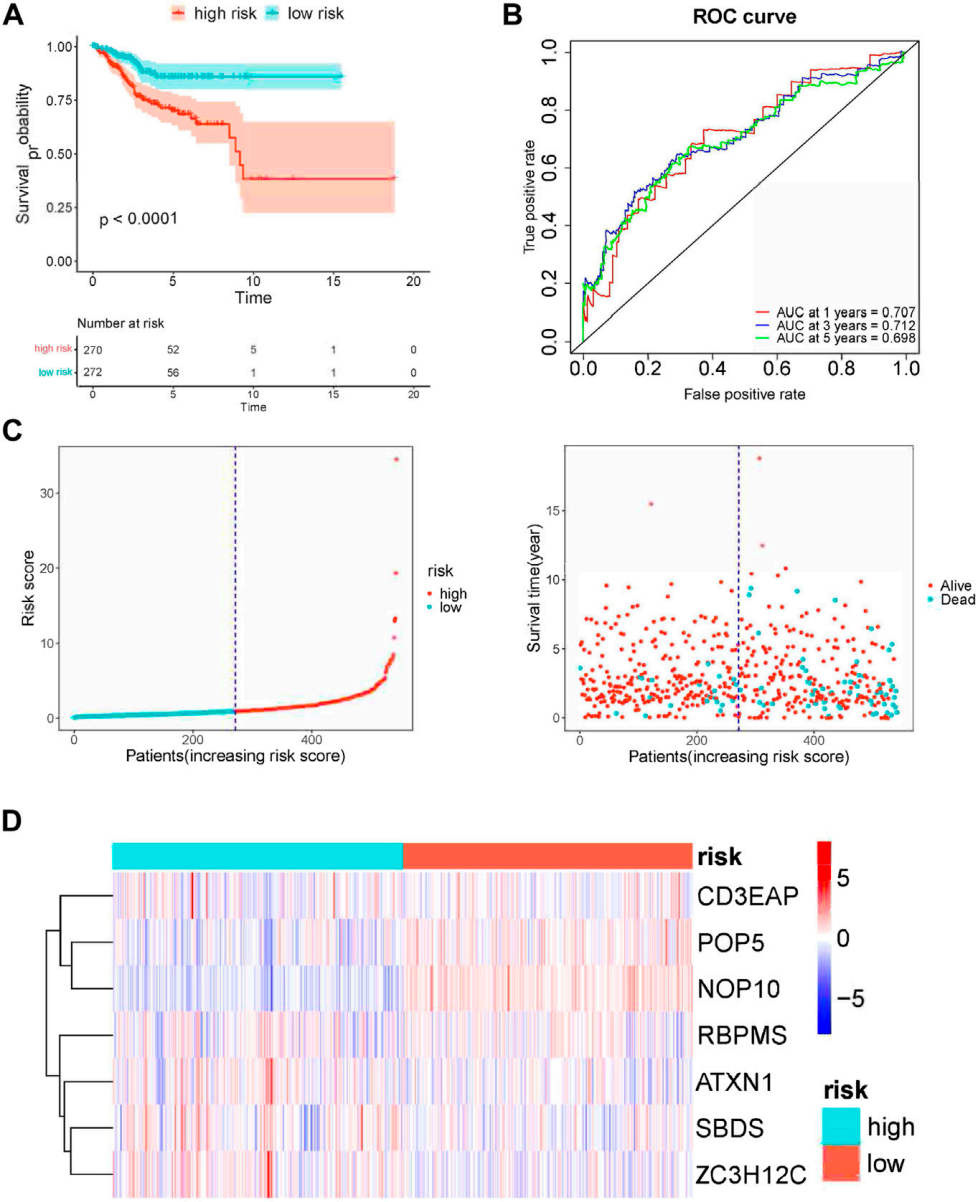
Risk score analysis of seven-gene prognostic model in TCGA UCEC cohort. **(A)** Kaplan–Meier curves of OS of high- and low-risk groups (*p* < 0.0001). **(B)** ROC curve for judging the accuracy of the signature. **(C)** The distribution of risk scores, gene expression levels and patient survival status. The dotted line represents the median cut point and divides patients into low-risk and high-risk groups. **(D)** The heatmap of the seven hub genes expression profiles in the group.

Then based on the median risk score as the cut-off point, UCEC patients were divided into low- (n = 272) and high-risk groups (n = 271). The K-M results showed that high-risk patients had significantly worse survival than low-risk patients (Figure 6A). Meanwhile, the AUC of the whole training set was 0.706 for 1-year, 0.712 for 3-years, and 0.698 for 5-years. This suggested good accuracy for the seven-gene signature (Figure 6B). We also calculated the 95% CI of AUCs values: the 95% CI of 1-year is 0.594–0.817, the 3-years 95% CI is 0.620–0.785, and the 5-years 95% CI is 0.608–0.777. The RFS status of patients and heatmap of expression profiles of these seven genes in the high- and low-risk groups are displayed (Figures 6C,D).

### Genetic alterations, methylation analysis, and immunohistochemical profiles of the hub RNA binding proteins

**FIGURE 7:**
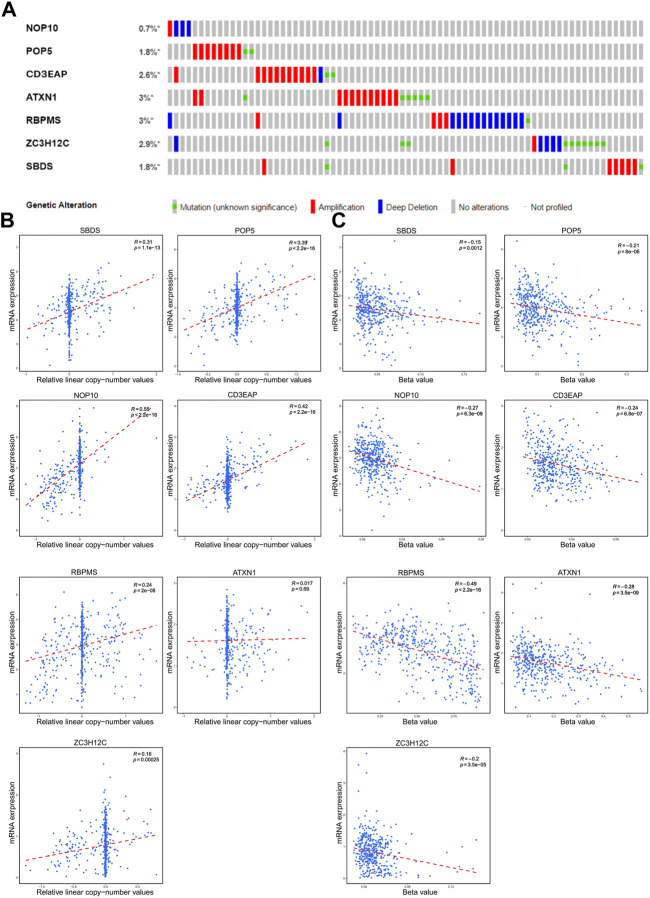
The genetic alterations and methylation of the seven hub RBPs **(A)** The genetic alterations of the seven genes. **(B)** The correlation between mRNA expression and copy number values of the seven genes. **(C)** The correlation between mRNA expression and DNA methylation of the seven genes.

**FIGURE 8:**
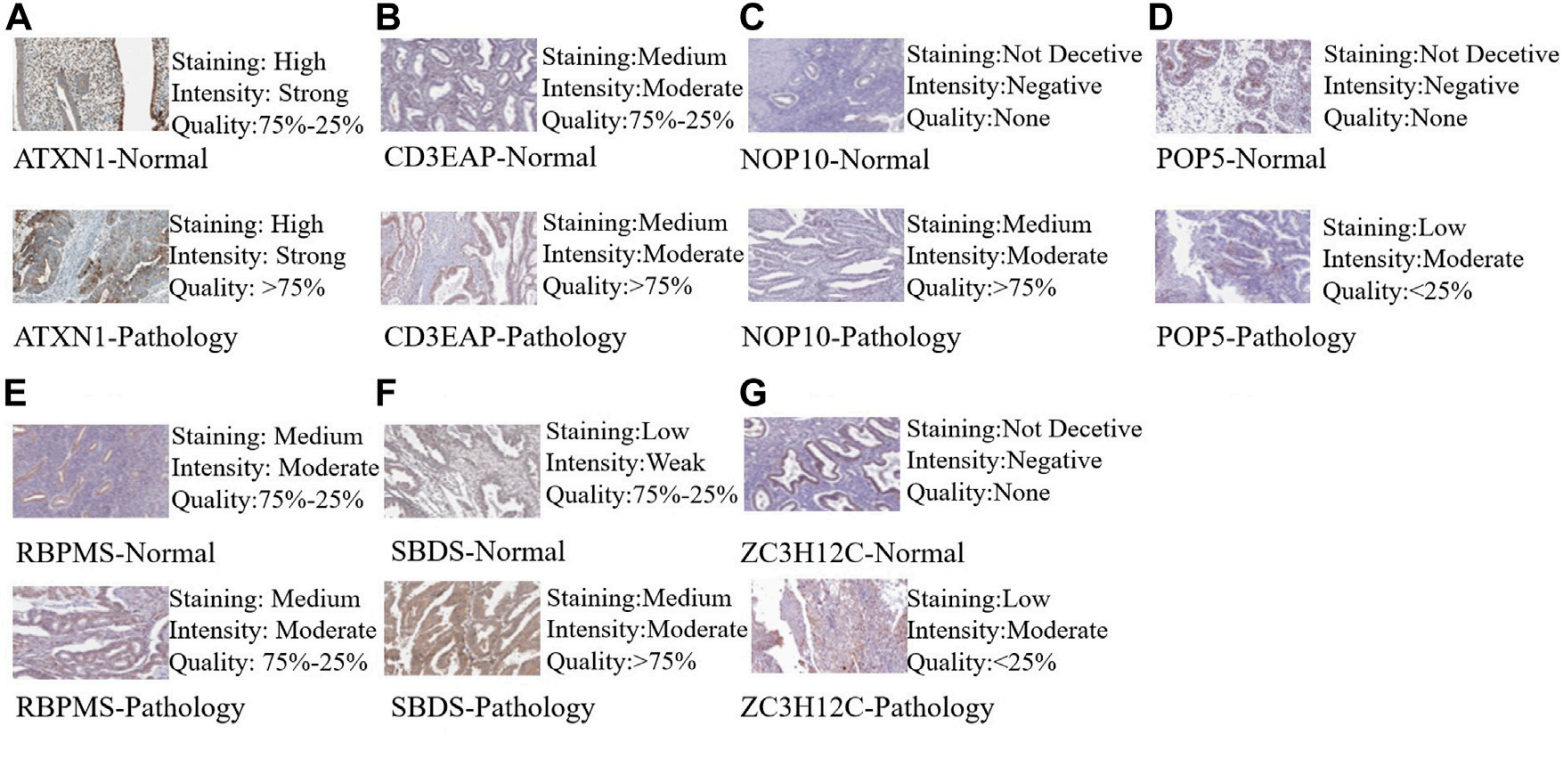
Validation of hub RBP expression in UCEC and normal tissue from the HPA database. **(A)** ATXN1, **(B)** CE3EAP, **(C)** NOP10, **(D)** POP5, **(E)** RBPMS, **(F)** SBDS, **(G)** ZC3H12C.

Based on the cBioportal database, the genetic alterations and methylation profiles of seven RBPs were explored. NOP10, POP5, CD3EAP, ATXN1, ZC3H12C, RBPMS, SBDS exhibited 0.7%, 1.8%, 2.6%, 3%, 2.9%, 3% and 1.8% of genetic changes, respectively (Figure 7A); most of these concerned copy number variation. The correlations between copy number variation and mRNA expression of SBDS, POPS, NOP10, and CD3EAP were 0.31, 0.37, 0.54, and 0.42, respectively (Figure 7B). In addition, the correlations between DNA methylation and mRNA expression of RBPMS and ATXN1 were -0.49 and -0.31, respectively (Figure 7C). These findings suggested that SBDS, POPS, NOP10, and CD3EAP might be copy-number drive genes. Similarly, RBPMS and ATXN1 might be methylation-drive genes, and ZC3H12C might be co-driven by copy-number and methylation or other regulatory factors. We further explored the protein expression levels of these hub RBPs in UCEC using immunohistochemical results from the HPA database. Compared with non-tumor tissues, NOP10, POP5, and CD3EAP expression levels were higher in UCEC tissues; the other four showed no significant difference (Figure 8).

### Immune infiltration analysis of hub RNA binding proteins

**FIGURE 9:**
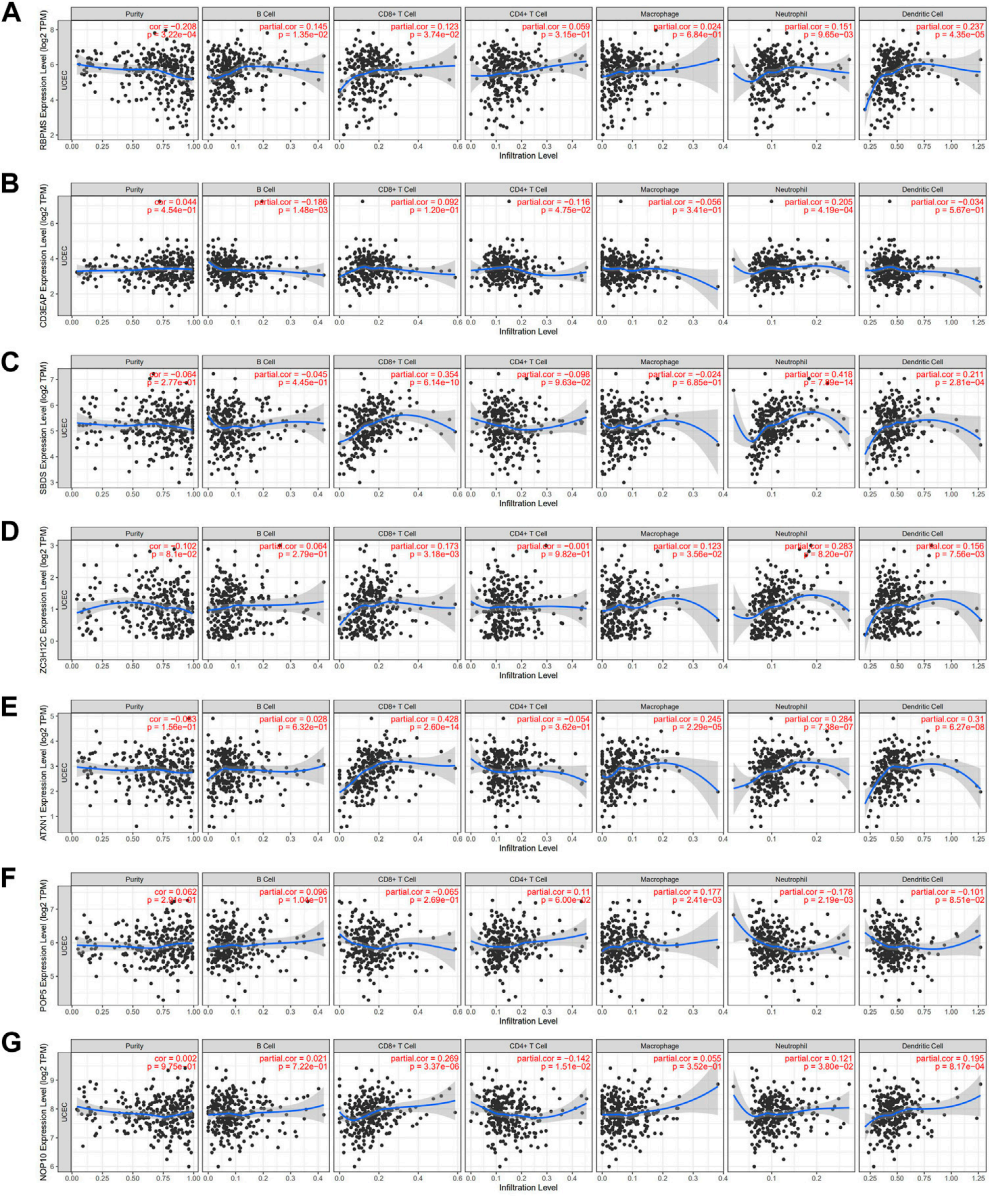
Relationships between expression of seven hub RBPs and tumor immune infifiltrations **(A)** RBPMS, **(B)** CE3EAP, **(C)** SBDS, **(D)** ZC3H12C, **(E)** ATXN1, **(F)** POP5, **(G)** NOP10.

Since immune cells participate in the formation of the tumor microenvironment, they significantly contribute to tumor development and prognosis. We studied the potential connection between seven hub RBPs and immune infiltration (purity, B cells, CD8+T cells, CD4+T cells, macrophages, neutrophils, and dendritic cells) in the UCEC. SBDS was positively correlated with CD8^+^ T cells (partial. Cor = 0.354, *p* = 6.14e-10), neutrophils (partial. Cor = 0.418, *p* = 7.89e-14) and dendritic cells (partial. Cor = 0.211, *p* = 2.81e-04). NOP10, ATXN1, and ZC3H12C also showed similar immune infiltration RBPMS was the only gene associated with infiltration purity (cor = -0.208, *p* = 3.22e-4), CD3EAP was the only gene associated with B Cell (partial. Cor = -0.186, *p* = 1.48e-03) (Figure 9).

### Correlations between the seven-RNA binding proteins prognostic model and clinical parameters

**FIGURE 10:**
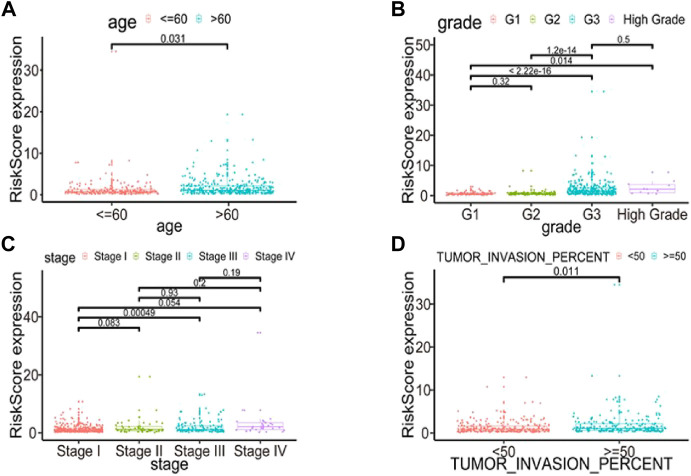
Relationships between risk score and various clinical parameters. Risk scores in cohorts stratified by age **(A)**, grade **(B)**, stage **(C)**, tumor invasion percent **(D)**. Risk score is significantly associated with age, grade and tumor invasion percent, but not completely with stage.

To further explore the model’s prognostic value, we investigated the relationships between the risk score and various clinical parameters (Figure 10). The risk score was significantly associated with age and grade; the higher the risk score, the greater percent of tumor invasion. However, this association was only present in stage I and III disease samples.

### Building a predictive, risk score-based nomogram

We performed univariate and multivariate Cox regression analyses based on RNA expression profiles and clinical information from TCGA data to determine whether the risk score was an independent prognostic factor. The univariate analysis showed that age, grade, stage, tumor invasion percent, and risk score were significantly correlated with OS in UCEC patients (Supplementary Figure S4A). Multivariate analysis indicated that age, grade, stage, tumor invasion percent, and risk score were all independent prognostic factors for OS (Supplementary Figure S4B). Thus, the risk score (based on seven RBPs) might serve as an independent prognostic factor for UCEC patient survival.

**FIGURE 11:**
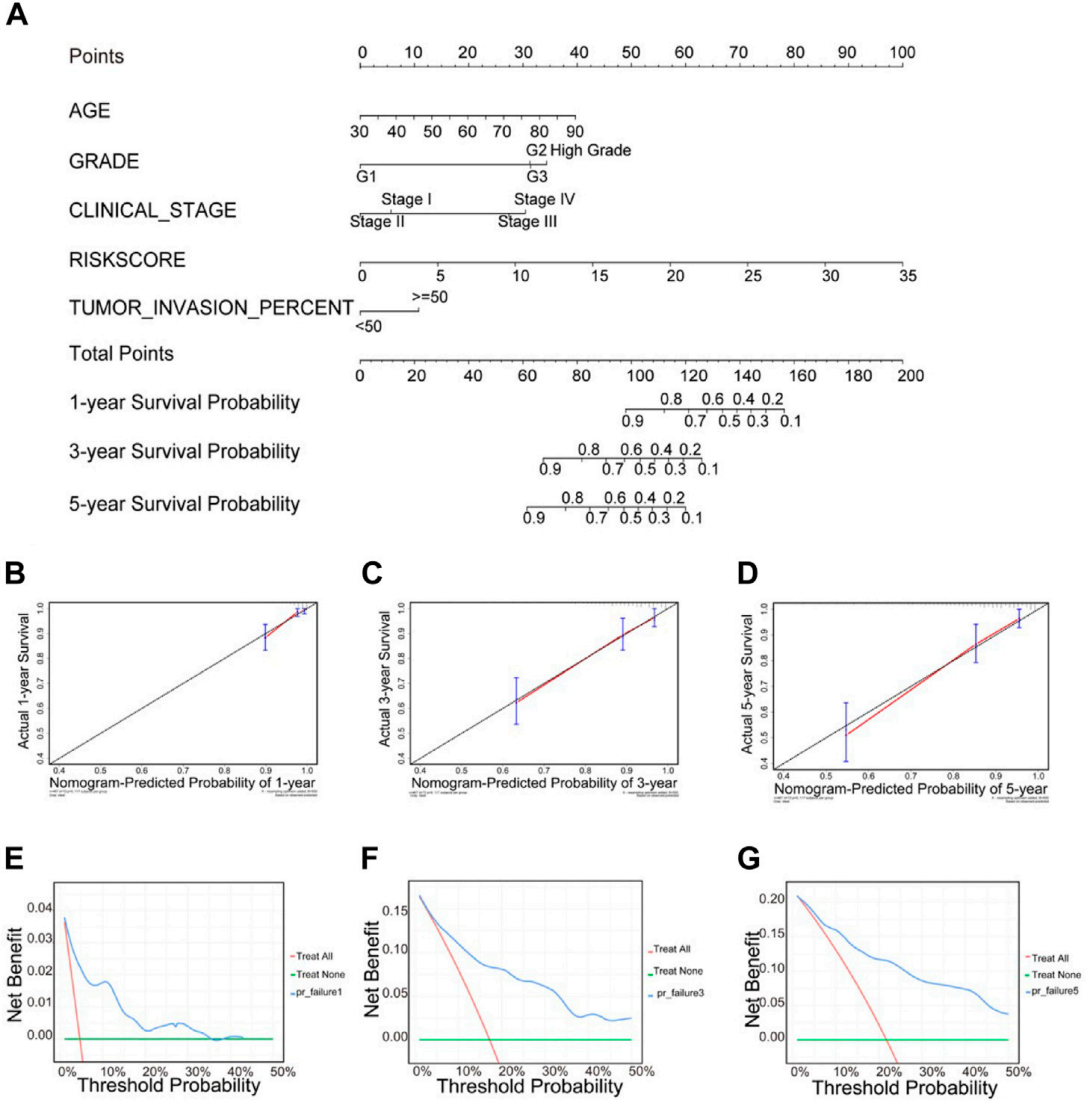
Clinical prognostic nomogram for predicting prognosis in TCGA UCEC cohorts. **(A)** The clinical nomogram developed to predict 1-year, 3-years and 5-years survival by incorporating five independent prognostic indicators, including risk score. Calibration curves showing nomogram predictions for one- year **(B)**, 3-years **(C)** and 5-years **(D)** survival. Decision curve analysis was used to estimate clinical usefulness and net benefit of the predictive nomogram for 1-year **(E)**, 3-years **(F)** and 5-years **(G)**.

The nomogram uses an algorithm that includes multiple variables to calculate the predicted probability of a patient reaching a specific clinical endpoint, quantifying the relative contribution of each risk factor, which can be a useful tool to present and help understand clinical prediction models (Irish et al., 2003; Park, 2018). Therefore, to develop a quantitative method for predicting prognosis in UCEC patients, we constructed a nomogram that integrated risk score and the independent predictors identified above (age, grade, tumor invasion percent, and stage). Each variable was assigned a score. Then, scores for the five variables were added, and a vertical line was drawn from the total score to the nomogram subscale to determine the estimated 1-, 3-, and 5-years survival rates (Figure 11A). The c-index value of the prediction nomogram was 0.816 for the UCEC cohort, indicating that it had good discrimination capability. The calibration curves indicated that the nomogram predictions were consistent with actual observations for 1-, 3- and 5-years OS in the TCGA-UCEC cohort, suggesting that the nomogram was reliable (Figures 11B–D). Additionally, DCA was used to evaluate the clinical efficiency of the predictive nomogram. The results showed that the nomogram could improve patient prognosis predictions (Figures 11E–G).

### Correlation of hub RNA binding proteins ’ expression level and patients’ risk scores

In addition, we analyzed the relationship between the expression value of each hub RBPs and the risk of UCEC patients. The results showed that 5/7 hub RBP (*ATXN1, CD3EAP, RBPMS, SBDS* and *ZC3H12C*) was significantly associated with the risk of patients, with respective *p*-values of 0.046, <2.2e-16, 0.016, 2.5e-07, and 0.0021 (Supplementary Figure S5).

### Validation of hub RNA binding proteins’ prognostic value

To determine the feasibility and reliability of the seven-gene prognostic signature, we validated it using testing set A (n = 272) and testing set B (n = 271). In the testing sets A and B, a lower overall survival rate was noted for patients in the high risk compared to those in the low risk groups (Supplementary Figure S6). The AUC for the testing set A and B were enough to indicate that the signature strongly predicts overall survival in UCEC patients.

## Discussion

Many studies have confirmed that differential expression of RBPs is closely associated with developing various tumor types (Pereira et al., 2017b; Neelamraju et al., 2018). However, there has been little information on the expression and potential role of RBPs in UCEC based on RNA-seq data from the TCGA cohort. Herein, 128 differently expressed RBPs were identified in tumor tissues compared to normal tissues based on RNA-seq data from the TCGA-UCEC cohort. We used GO and KEGG enrichment analyses to elucidate the underlying biomolecular mechanism of differently expressed RBPs. Then, the PPI network was constructed by STRING and visualized by Cytoscape. Furthermore, univariate Cox regression and multiple stepwise Cox regression of hub RBPs were performed to further explore their potential value in clinical outcomes. Finally, a risk model was developed to predict the prognosis of UCEC based on seven RBP genes. These findings facilitate the identification of new biomarkers for the diagnosis and prognosis prediction of UCEC.

The seven hub RBPs *CD3EAP, POP5, NOP10, ATXN1, ZC3H12C, RBPMS,* and *SBDS* were implicated in the progression and prognosis of many cancers. Studies have shown that CD3EAP affects the transcription process of rRNA, RNA polymerase activity, and cell proliferation; CD3EAP can also mediate the activation pathway of T cells to produce leukocyte interleukin-2 to inhibit the growth of cancer cells (Lovci et al., 2016; Wang et al., 2019). Haoya Xu et al. found that the high expression of *CD3EAP* in endometrial cancer was related to a higher pathological grade, later clinical stage, and postoperative tumor recurrence (Xu et al., 2021a). *CD3EAP* is among the genes with the highest mutation rate in hepatoid adenocarcinoma of the stomach (Baralle and Giudice, 2017). Noncoding transcripts *POP5* plays a significant role in the processing of tRNA (Hazeyama et al., 2013) and studies have shown that it is a new biomarker for prostate cancer (Romanuik et al., 2009). ATXN1 can induce RNA degradation and translation suppression by binding to the 5’ non-translation zone of miR760 (Nitschke et al., 2020). Ribonucleoprotein NOP10 is involved in pathways related to rRNA processing in the nucleus and cytosol and gene expression. Loss of NOP10 and subsequent reduction of H/ACA box snoRNAs and rRNA pseuduridylation inhibited the formation, migration, and invasion of lung cancer cell growth colonies (Cui et al., 2021). *Zinc Finger CCCH-Type Containing 12C* (*ZC3H12C*) is a protein-coding gene that may act as an RNase and regulate levels of target RNA species (Ota et al., 2004; Taylor et al., 2006). Some studies found it a prognosis-related center for lung adenocarcinoma RBP (Liu, 2020). RNA-binding protein with multiple splicing (RBPMS) is a higher vertebrate mRNA-binding protein containing a single RNA recognition motif (RRM). RBPMS have been proved to be involved in mRNA transport location and stability and play a key role in axon-guided smooth muscle plasticity and regulation of cancer cell proliferation and migration (Sun et al., 2006; Teplova et al., 2016). Fusion involving the neuromodulin-1 gene (*NRG1*) leads to ERBB-mediated pathway activation, thus providing a reasonable candidate for targeted therapy. Multiple NRG1 fusion partners, including RBPMS, have been found in lung cancer patients. *SBDS* (Laskin et al., 2020) encodes a highly conserved protein essential in ribosome biogenesis. *SBDS* is often overexpressed or amplified in human cancers, and high endogenous *SBDS* are significantly associated with adverse prognoses. In contrast, *SBDS* knockdown leads to the stabilization and activation of p53 through the ribosomal stress-RPL5/RPL11-MDM2 pathway, inhibiting cancer cell proliferation and invasion (Hao et al., 2020).

Aberrant DNA methylation leads to malignancy, mainly through DNA hypermethylation or hypomethylation (Heyn and Esteller, 2012). This is reflected in individual genes and genome-wide (Pan et al., 2018). An increased number of DNA hypomethylation has been shown to activate oncogenes and affect chromosome stability and several retrotransposon elements (Ahuja et al., 2016). We found that multiple methylation sites of regulated genes in UCEC were negatively correlated with their own expression. These data suggest that methylation changes may lead to abnormal gene expression, confirming that alterations in DNA methylation can be exploited in cancer diagnosis (Esteller, 2007).

In order to further explore the role of these hub RBPs in the progression of UCEC. We searched for differently expressed genes between high and low risk patients, and performed GO analysis to explore their possible role in the development of cancer (Supplementary Figure 7). The results showed that in the aspect of BP, these differently expressed genes were significantly enriched in cell development (p-adjusted value = 5.19e-8), Cell differentiation (p-adjusted value = 1.93e-7) and cell-cell adhesion *via* plasma-membrane adhesion molecules (p-adjusted value = 4.01e-5), which are common pathways affecting cancer. In the aspect of KEGG, we noted that these differently expressed genes significantly affect the function of Cytochrome P450 (p-adjusted value = 0.019), which is mainly distributed in the endoplasmic reticulum and mitochondrial inner membrane, and plays an important role in both cytokines and thermoregulation (Guengerich, 2019). Tumor cells depend on glycolysis and mitochondrial oxidative phosphorylation for survival (Hanahan and Weinberg, 2000). Abnormal mitochondrial pathways and metabolic disorders can lead to altered gene expression that promotes cancer progression and immune system evasion. In addition, a large number of enrichment pathways are involved in membrane and cell signal transduction (gated channel activity, p-adjusted value = 3.80e-9; synapse, p-adjusted value = 1.19e-11; receptor ligand activity, p-adjusted value = 8.49e-6), which may influence the occurrence of tissue inflammation or insensitivity to antigrowth signals, and thus affect the progression of cancer (Hanahan and Weinberg, 2000).

Whether confined to specific genes or affecting the entire chromosome, changes in DNA copy number have been implicated in disease and developmental abnormalities and as a source of adaptive potential. As the gain or loss of genetic information affects gene expression, large-scale changes in gene copy numbers have profound effects on the protein composition of cells (Tang and Amon, 2013). Epidermal growth factor receptor copy number alterations have been associated with poor clinical outcomes in patients with head and neck squamous carcinoma (Temam et al., 2007). Increased hub RBP copies were positively correlated with UCEC expression. Future studies should continue to search for new biomarkers of UCEC.

Immune infiltration is an important factor affecting cancer prognosis (Balachandran et al., 2015). Most of the seven hub RBPs have no correlation with tumor purity except RBPMS. Notably, all prognostic RBPs were positively associated with neutrophil infiltration. Studies have shown that tumor-associated macrophages and neutrophils perform pretumor cell functions, enhancing tumor cell invasion, metastasis, angiogenesis, and extracellular matrix remodeling while inhibiting anti-tumor immune monitoring (Xu et al., 2021b). RBPs are likely involved in cancer invasion and metastasis; these mechanisms require further study.

This was the first study to link molecular markers to endometrial cancer, closely associate differently expressed genes with the clinicopathological features of endometrial cancer, and construct a risk model and nomogram. This nomogram could be deployed in clinical settings to provide an intuitive and convenient tool for individualized diagnosis and treatment of endometrial cancer. The inclusion of potential biomarkers based on the molecular level would make the nomogram predictions more general and accurate. However, the nomogram’s accuracy might decrease over time due to improvements in treatment, early detection, and changes in natural history (Balachandran et al., 2015); therefore, shortcomings remain. In addition, our results should be considered in the context of several study limitations. Firstly, the prognostic information of endometrial cancer patients was not available in other databases such as GEO and ICGC, which we could not validate using external datasets (Xu et al., 2021b), and some important clinical characteristics of UCEC patients, such as living environment and family history, were missing from the TCGA database, thus more uterine endometrial samples from multicenter and detailed clinical information are necessary for validation. Secondly, *in vitro* and *in vivo* experiments are needed to clarify the molecular mechanisms for better clinical applications in further studies.

This study systematically analyzed a large cohort of patients in the TCGA database. This allowed us to construct a robust prognostic model based on seven RBPs, which may be of great value in clinical applications. In addition, our prognostic model can also accurately predict survival when patients are stratified into different cohorts based on other disease characteristics. The AUC at 1 year, 3 years is greater than 0.7 and the AUC at 5 years is also very close to 0.7, which indicates the model’s evaluation power with moderate performance (Huang and Ling, 2005). Moreover, We constructed a nomogram by integrating the seven RBPs model and clinical information to improve the accuracy of prediction and clinical application value. Our signature may therefore help physicians make accurate, individualized survival predictions. In conclusion, we performed a comprehensive bioinformatics analysis based on TCGA data to investigate the prognostic value of aberrantly expressed RBPs in UCEC patients. We expect our work will facilitate the future development of new diagnostic and therapeutic strategies for UCEC and inform future investigations.

## Conclusion

We constructed a prognostic-associated risk model of UCEC patient survival based on the expression of seven hub RBPs within tumor tissues. Our novel signature can be used as an additional clinical tool to facilitate personalized therapy in UCEC patients. While this model exhibited significant prognostic value, this study is limited by the fact that it is solely based upon data within the TCGA database and lacks any external validation. If the model wants to be further improved in the future, we need more external data sets to confirm and expand upon our findings.

## Data Availability

The original contributions presented in the study are included in the article/Supplementary Material, further inquiries can be directed to the corresponding author.

## Abbreviations

RBPs, RNA binding proteins; UCEC, Uterine Corpus Endometrial Carcinoma; TCGA, The Cancer Genome Atlas database; HPA, The Human Protein Atlas; BMI, body mass index; EMT, epithelial-mesenchymal transition; CNV, copy number variation; K-M, Kaplan-Meier; AUC, Area Under Curve; c-index, consistency index; OS, overall survival; DCA, decision curve analysis; BP, biological process; CC, cellular component; MF, molecular function; HR, hazard risk; RRM, RNA recognition motif.
