# Supplementary figures and images for: Comprehensive landscape of the functions and prognostic value of RNA binding proteins in uterine corpus endometrial carcinoma
Source: Front Mol Biosci. 2022 Oct 3;9:962412. doi: 10.3389/fmolb.2022.962412 (PMC9574853; doi:10.3389/fmolb.2022.962412)

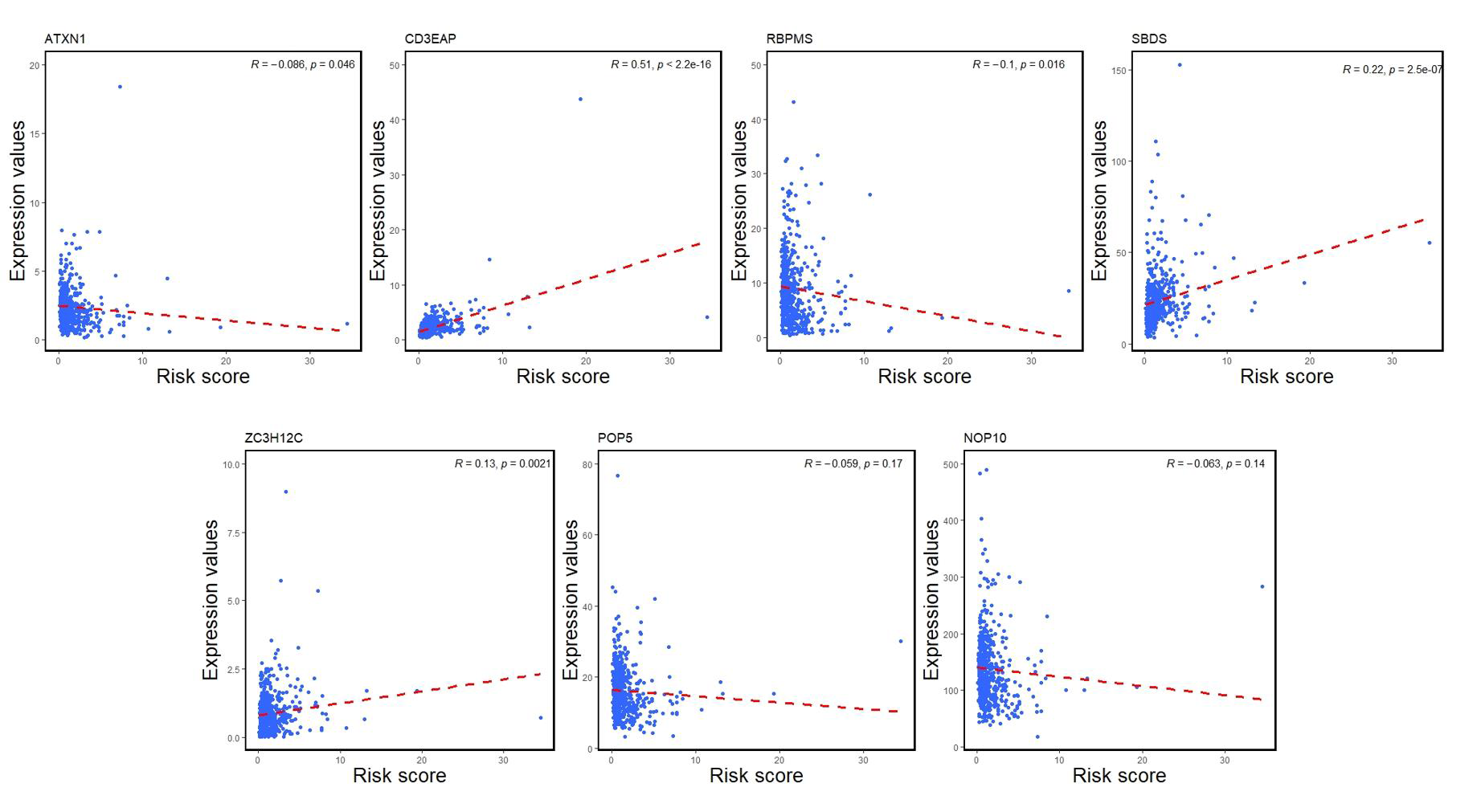

Supplement: Supplementary file 1 [file Image5.PNG]

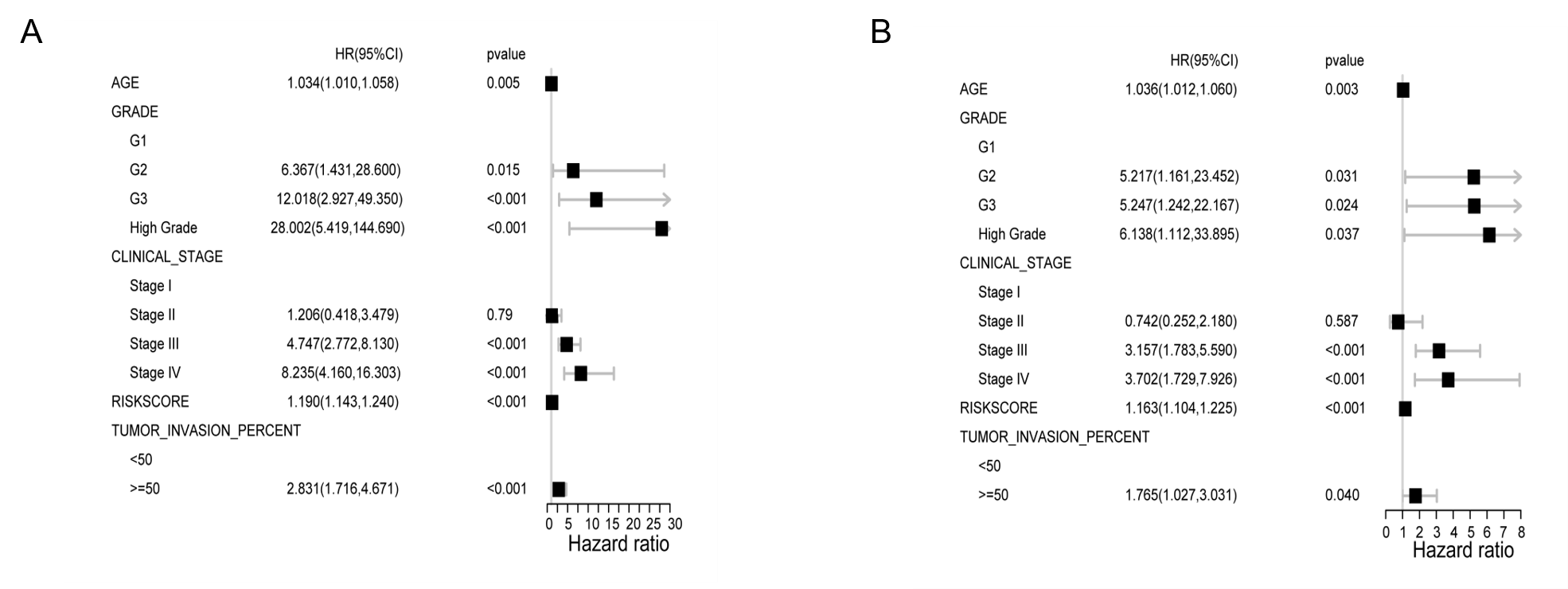

Supplement: Supplementary file 2 [file Image4.PNG]

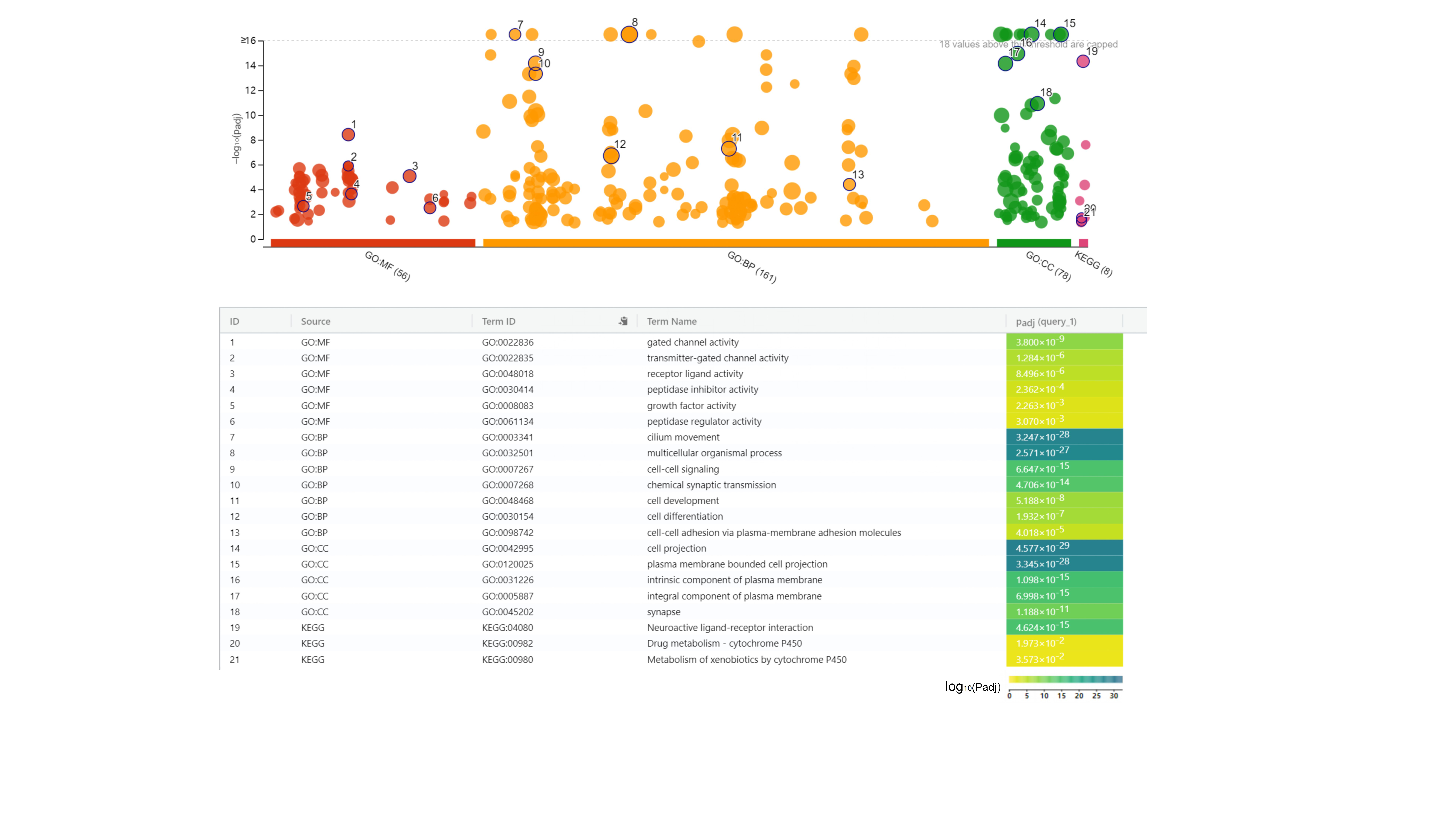

Supplement: Supplementary file 3 [file Image7.PNG]

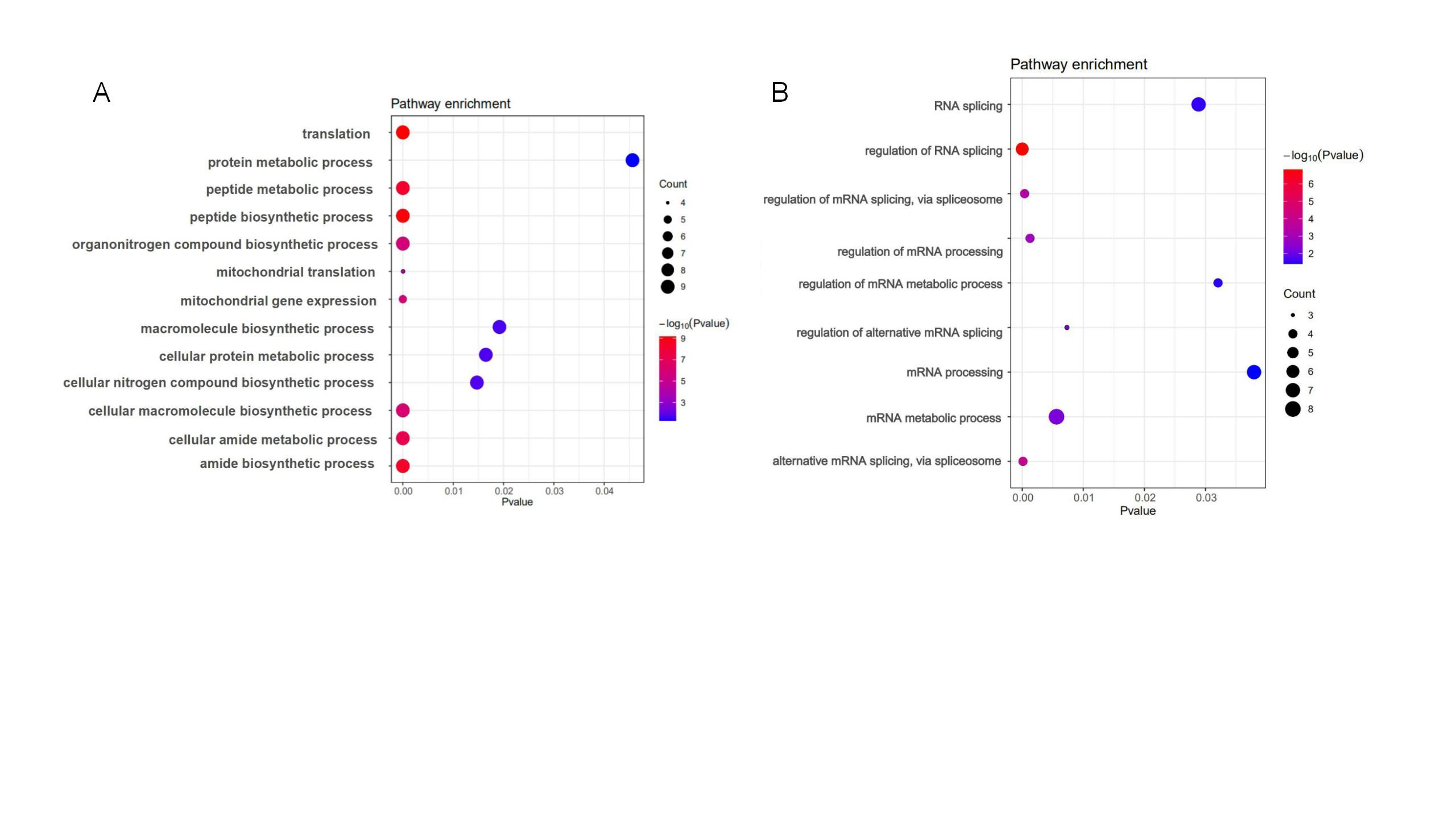

Supplement: Supplementary file 4 [file Image2.PNG]

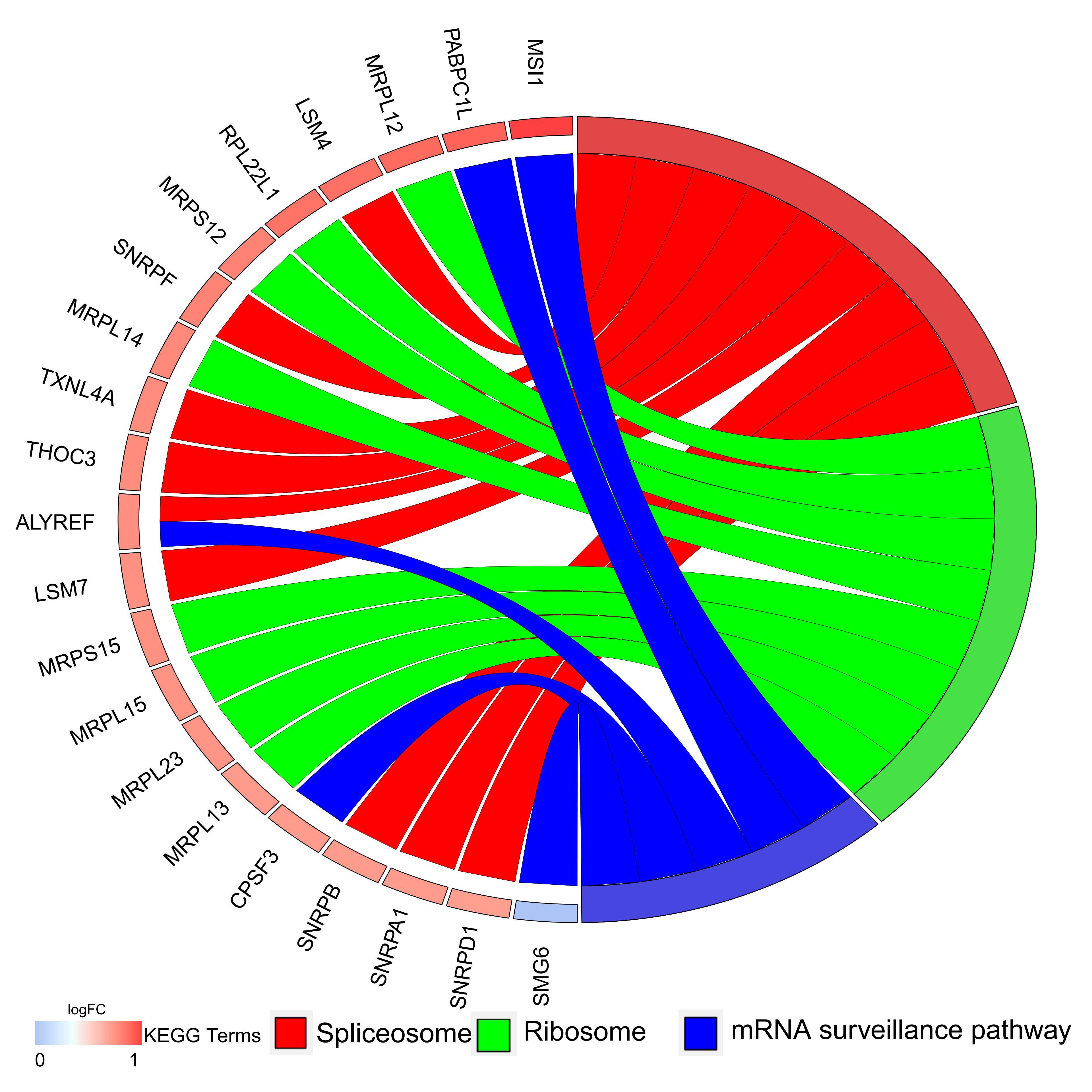

Supplement: Supplementary file 5 [file Image1.PNG]

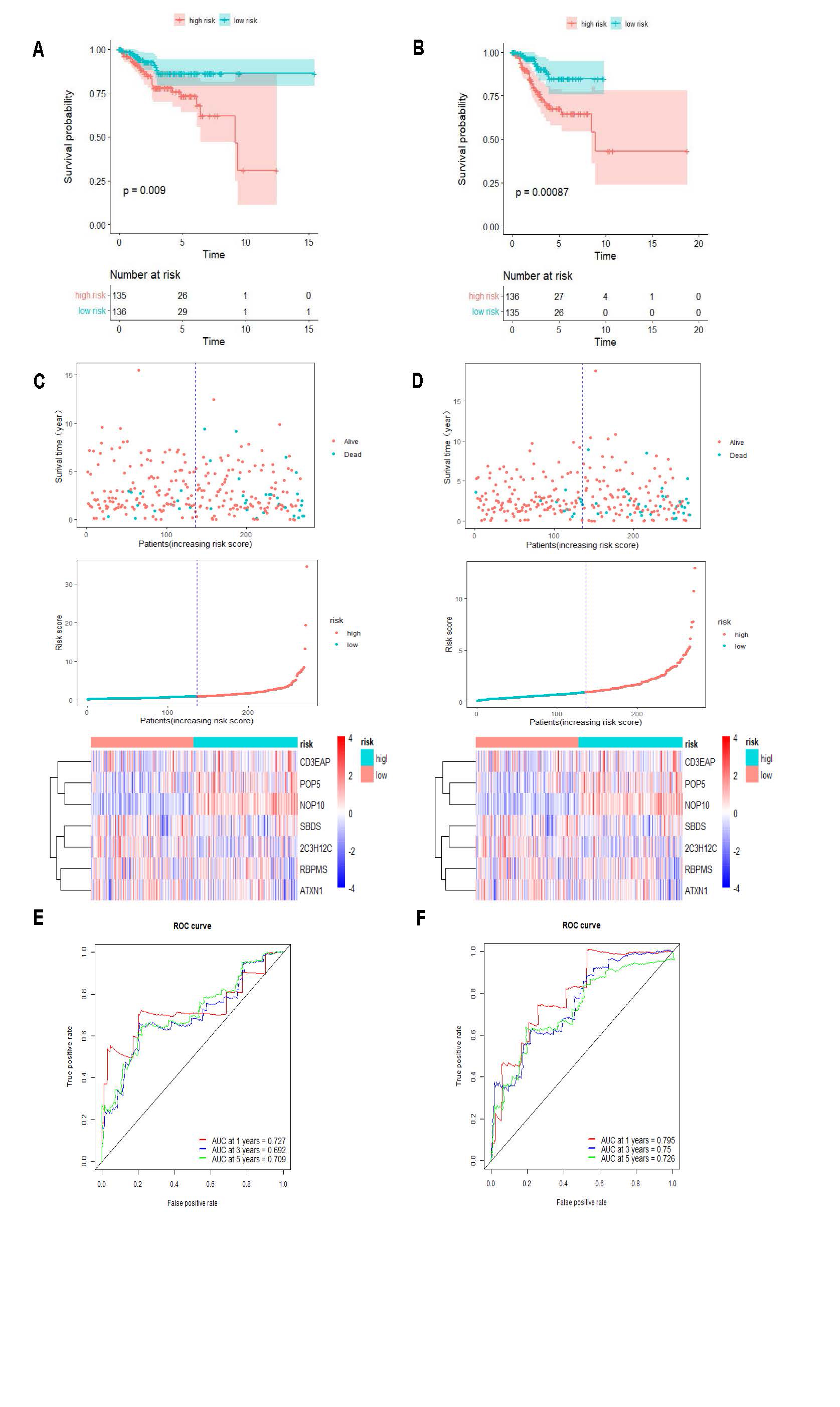

Supplement: Supplementary file 6 [file Image6.PNG]

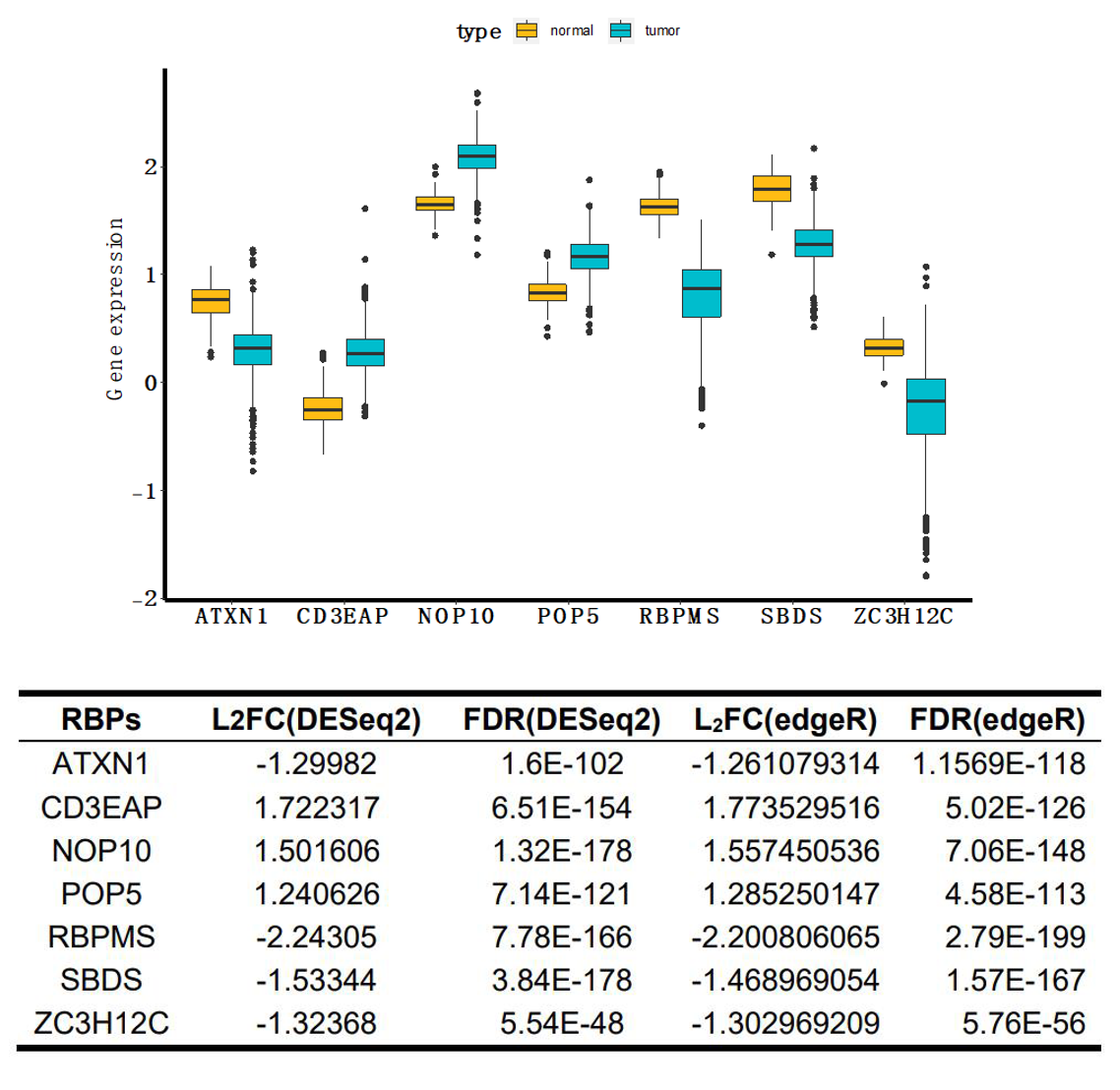

Supplement: Supplementary file 7 [file Image3.PNG]
